# Serologic Response and Safety after a Third Dose of the COVID-19 BNT162b2 Vaccine in Patients with Inflammatory Bowel Diseases

**DOI:** 10.3390/vaccines11071263

**Published:** 2023-07-20

**Authors:** Hadar Edelman-Klapper, Keren Masha Rabinowitz, Eran Zittan, Ariella Bar-Gil Shitrit, Idan Goren, Irit Avni-Biron, Jacob E. Ollech, Lev Lichtenstein, Hagar Banai-Eran, Henit Yanai, Yifat Snir, Maor H. Pauker, Adi Friedenberg, Adva Levy-Barda, Yelena Broitman, Haim Ben Zvi, Tsachi-Tsadok Perets, Rami Eliakim, Revital Barkan, Sophy Goren, Dani Cohen, Iris Dotan

**Affiliations:** 1 Division of Gastroenterology, Rabin Medical Center, Petah Tikva 4941492, Israel; hadared@clalit.org.il (H.E.-K.); kerenra3@clalit.org.il (K.M.R.); goreni@ccf.org (I.G.); iritab@clalit.org.il (I.A.-B.); jacobel@clalit.org.il (J.E.O.); agarb@clalit.org.il (H.B.-E.); henitya@clalit.org.il (H.Y.); yifats3@clalit.org.il (Y.S.); maorha11@clalit.org.il (M.H.P.); adifr2@clalit.org.il (A.F.); broytman@clalit.org.il (Y.B.); revitalba11@clalit.org.il (R.B.); 2 Sackler Faculty of Medicine, Tel Aviv University, Tel Aviv 6997801, Israel; haimbe@clalit.org.il (H.B.Z.); abraham.eliakim@sheba.health.gov.il (R.E.); 3 Felsenstein Medical Research Center, Sackler School of Medicine, Tel Aviv 6997801, Israel; 4 The Abraham and Sonia Rochlin IBD Unit, Department of Gastroenterology, HaEmek Medical Center, Faculty of Medicine, Israel Institute of Technology, Afula 1834111, Israel; eranzittan@clalit.org.il; 5 Rappaport Faculty of Medicine Technion-Israel Institute of Technology, Haifa 3109601, Israel; 6 IBD MOM Unit, Shaare Zedek Medical Center, Digestive Diseases Institute, Jerusalem 9103102, Israel; ariellash@szmc.org.il; 7 Faculty of Medicine, Hebrew University of Jerusalem, Jerusalem 9112102, Israel; 8 Clalit Health Services, Petah Tikva 4933355, Israel; levli@clalit.org.il; 9 Adelson School of Medicine, Ariel University, Ariel 4077625, Israel; 10 Biobank, Rabin Medical Center, Department of Pathology, Petah Tikva 4941492, Israel; advale11@clalit.org.il; 11 Microbiology Laboratory, Rabin Medical Center, Petah Tikva 4941492, Israel; 12 Gastroenterology Laboratory, Division of Gastroenterology, Rabin Medical Center, Petah Tikva 4941492, Israel; tzahipe1@clalit.org.il; 13 Holon Institute of Technology, Department of Digital Medical Technologies, Holon 5810201, Israel; 14 Department of Gastroenterology, Sheba Medical Center, Ramat Gan 52621, Israel; 15 School of Public Health, Sackler Faculty of Medicine, Tel Aviv University, Tel Aviv 6997801, Israel; sophyg@tauex.tau.ac.il (S.G.); dancohen@tauex.tau.ac.il (D.C.)

**Keywords:** COVID-19, anti-TNFα, inflammatory bowel diseases, BNT162b2 vaccine

## Abstract

Vaccines are pivotal for control of the coronavirus disease (COVID-19) pandemic. Patients with inflammatory bowel diseases (IBDs) treated with antitumor necrosis factor (TNF)-α have lower serologic response after two COVID-19 vaccine doses. Data regarding a third vaccine dose are scarce. An Israeli multicenter prospective observational study recruited 319 subjects: 220 with IBD (79 treated with anti-TNFα) and 99 healthy control (HC) participants. All patients received two mRNA-BNT162b2 vaccines (Pfizer/BioNTech), 80% of whom received a third vaccine dose. Evaluation included disease activity, anti-spike (S) and nucleocapsid (N) antibody levels, anti-TNFα drug levels, and adverse events (AEs). All participants showed significant serologic response one month after receiving a third dose. However, three months later, the anti-S levels decreased significantly in patients treated with anti-TNFα compared with the non-anti-TNFα and HC groups. A correlation between serologic response to the third vaccine dose and anti-TNF drug levels was not found. No significant AE or IBD exacerbation was observed. Importantly, lower serologic response after the third vaccine dose predicted infection. A third dose of BNT162b2 is effective and safe in patients with IBD. Lower serologic response predicted infection, even in seropositive subjects. Lower serologic responses and their rapid decline suggest a fourth vaccine dose in this patient population.

## 1. Introduction

Vaccination contributed to overcoming coronavirus disease (COVID-19) pandemic. Since antibody response to COVID-19 vaccines decreases over time [1,2,3,4,5], many countries, Israel included, recommended a third vaccine dose at least 6 months after the second dose [6]. Several studies have demonstrated that a third vaccine dose is effective and safe and reduces mortality [7,8], specifically in the older population [5,9]. However, response to the third dose in immunocompromised individuals or those treated with antitumor necrosis factor (TNF)-α has scarcely been reported.

We previously highlighted the need for a third vaccine booster dose in patients with inflammatory bowel diseases (IBDs) treated with anti-TNF-α due to the sharper decline in their serologic response compared to patients not treated with anti-TNFα and healthy controls (HCs) [3,10,11,12,13].

In this study, we prospectively followed patients with IBD stratified according to medical treatment one and three months after the third BNT162b2 vaccine dose, evaluating both efficacy and safety.

## 2. Materials and Methods

### 2.1. Design and Participants

This study was based on our previous work, as described in (K. M. Rabinowitz et al., ‘Anti-TNFα Treatment Impairs Long-Term Immune Responses to COVID-19 mRNA Vaccine in Patients with Inflammatory Bowel Diseases’, Vaccines (Basel), vol. 10, no. 8, p. 1186, Aug. 2022, doi:10.3390/VACCINES10081186/S1) [3]. Herein, we describe the serological response to the third BNT162b2 dose. The third vaccine dose was administered according to the Israeli Ministry of Health recommendation (30 July 2021) [6]. All patients were followed up to one year after vaccination, and >80% of patients received the third vaccine dose (30 µg) at least 6 months after the first dose. Several patients were recruited who received a third vaccine dose at least 6 months after the first vaccination without having previously participated in our study. The study was approved by the local IRBs of Rabin, Shaare Zedek, Emek, and Soroka Medical Centres (1072-20-RMC, 0557-20-SZMC, 0247-20-EMC, and 0568-20-SOR, respectively; MOH number: 2020-12-30_009617). All participants signed an informed consent form prior to enrollment in the study.

### 2.2. Study Procedure

**Figure 1:**
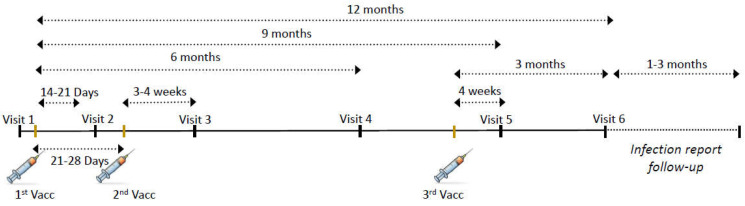
Study protocol. Patients were enrolled at visit 1, before the first vaccine dose. Visit 2 was 14–21 days after the first dose but before the second vaccine dose. Visits 3 and 4 were 1 and 6 months after the second vaccine dose, respectively. Visit 5 was 1 month after the third vaccine dose and ~9 months after the first dose. Visit 6 was 3 months after the third vaccine dose and 1 year after the first dose. In each visit, laboratory tests were performed, and questionnaires regarding disease severity and adverse events (AEs) were completed.

Participants who met the criteria were assessed six times. Visits occurred (i) prior to the first dose of the vaccine (visit 1), (ii) 14–21 days after the first and before the second dose of the vaccine (visit 2), (iii) 21–35 days after the second dose of the vaccine (visit 3), (iv) six months after the first dose (visit 4), (v) one month after the third dose (visit 5), and (vi) three months after the third dose (visit 6) (Figure 1). At nine months and a year after the initial vaccination (visits 5 and 6, respectively), subjects who rejected the third dose of the vaccine continued to be prospectively followed. Patients’ baseline demographics and IBD characteristics were evaluated at enrollment. The details of medical treatment, including duration and dosage, were recorded. Utilizing IBD-specific questionnaires like the Harvey–Bradshaw Index (HBI) [14] and the Simple Clinical Colitis Activity Index (SCCAI) [15], clinical evaluations were conducted during each visit. We proactively asked subjects about their symptoms and COVID-19 infection.

Laboratory methods and statistical analysis were performed as described in (K. M. Rabinowitz et al., ‘Anti-TNFα Treatment Impairs Long-Term Immune Responses to COVID-19 mRNA Vaccine in Patients with Inflammatory Bowel Diseases’, Vaccines (Basel), vol. 10, no. 8, p. 1186, Aug. 2022, doi: 10.3390/VACCINES10081186/S1) [3], including quantitative assays of SARS-CoV-2 IgG II, SARS-CoV-2 nucleocapsid (N)-IgG, Anti-TNFα drug, and antidrug antibodies.

## 3. Results

### 3.1. Study Population

The cohort included a total of 319 subjects recruited by four medical centers in Israel. Short and six-month responses of the cohorts to the first two vaccine doses were previously reported [3,10]. A total of 241 subjects of this cohort were further evaluated at visit 5, i.e., 1 month after receiving the third vaccine dose (*n* = 198) or 9 months after first vaccine dose in subjects declining to receive the third vaccine dose (*n* = 43; provided reasons: personal choice or previous infection). Israel’s first Omicron infection was reported in November 2021, and the wave began to decline in January 2022. Samples for visit 5 and visit 6 were collected between September 2021 and December 2021 and between December 2021 and March 2022, respectively. Of the 198 subjects vaccinated three times, 125 were patients with IBD, and 73 were HCs. Among the IBD group, 53 were treated with anti-TNFα agents (anti-TNFα group), and 72 were treated with other medical treatment or no medical treatment at all (non-anti-TNFα group). Baseline characteristics are detailed in Table 1. Subjects were examined 34 (29–46) (median [IQR]) days after the third vaccine dose and 267 (250–278) days after first vaccine dose. The median time between the second and third vaccine doses was 201 (187–216) days.

A total of 231 subjects were examined at visit 6, three months after third vaccine dose (*n* = 188) or one year after first vaccine dose in subjects declining a third dose (*n* = 43). Of the 188 subjects vaccinated three times, 112 were patients with IBD, and 76 subjects were HCs. Among the IBD group, 46 were in the anti-TNFα group, and 66 were in the non-anti-TNFα group. Baseline characteristics are detailed in Table 1. Patients were examined 134 (118–151) (median [IQR]) days after the third vaccine dose and 360 (354–369) days after first vaccine dose in those declining a third dose.

### 3.2. Patients with IBD Treated with Anti-TNFα Have Lower Serologic Responses to the Third Dose of COVID-19 BNT162b2 Vaccine

**Figure 2:**
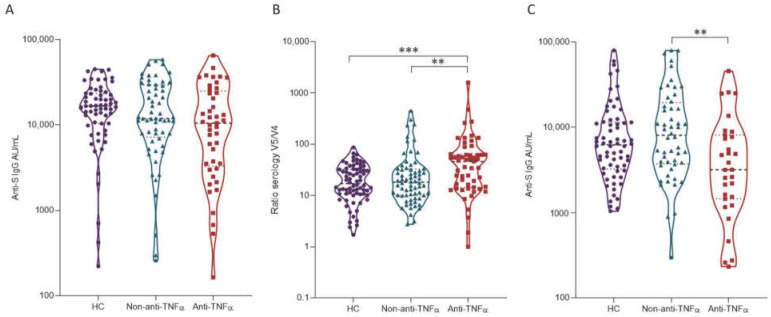
Serologic response one and three months after three doses of the BNT162b2 vaccine. (**A**) Serum anti-S levels of healthy controls (HC; purple circles), patients with IBD receiving non-anti-TNFα treatment (non-anti-TNF; blue triangles), and patients with IBD receiving anti-TNFα treatment (anti-TNF; red squares) at visit 5. Antibodies were quantified using the Abbott quantitative anti-S IgG kit. (**B**) Ratio between anti-S levels at visits 5 and 4 (V4; three months after two doses of the vaccine). (**C**) Serum anti-S levels of healthy controls (HC; purple circles), patients with IBD receiving non-anti-TNFα treatment (non-anti-TNF; blue triangles), and patients with IBD receiving anti-TNFα treatment (anti-TNF; red squares) at visit 6. Antibodies were quantified using the Abbott quantitative anti-S IgG kit. Statistical analysis was carried out using independent-samples Kruskal–Wallis test **—*p* < 0.01. *** *p* < 0.001. The black solid line denotes the median, and the black dashed lines denote IQR25-75.

We previously showed that anti-S levels were positive in all subjects a month after a second vaccine dose (visit 3) [10]. However, anti-TNFα treatment was associated with significantly lower anti-S levels. Furthermore, a steeper reduction in anti-S levels was observed six months after two vaccine doses in the anti-TNFα group [3]. At visit 5, one month after the third vaccine dose, a significant rise in anti-S levels was observed in all subject groups compared to pre-vaccination anti-S levels at visit 4 (Figure 2A). Interestingly, anti-S levels were still slightly lower in the anti-TNFα group compared to the non-anti-TNFα and HC groups; however, the difference between the groups was smaller than its magnitude after previous vaccinations. Moreover, the ratio between visit 5 and visit 4 was significantly higher in patients in the anti-TNFα group compared to the non-anti-TNFα and HC groups (*p* = 0.0028 and *p* = 0.0007, respectively; Figure 2B), suggesting a higher vaccine-boosting effect.

Anti-S levels were comparable between all treatments in the non-anti-TNFα group, further supporting our decision to include them in the same group—specifically, treatment with 5ASA (*n* = 19), vedolizumab (*n* = 18), no medical treatment (*n* = 19), and all other IBD medications (steroids (*n* = 2), immunomodulators (*n* = 2), ustekinumab (*n* = 9), and JAK inhibitors (*n* = 4); see Table 1, Figure S1).

Interestingly, three months after the third vaccine dose (visit 6), anti-S levels were significantly lower in the anti-TNFα group compared to the non-anti-TNFα group (Figure 2C; *p* = 0.0073). These results correspond to the observations following two vaccine doses [3,10] and support a steeper decline in vaccine effect [2,16,17].

As breakthrough infections were reported after mRNA vaccinations [18,19], we next asked whether subjects in our cohort were infected by SARS-CoV-2. Anti-N titers reflecting infection (and not vaccination) were positive one month after the third dose in only four subjects, all of whom were patients with IBD. Of those, two were treated with anti-TNFα (adalimumab and infliximab), and two were not (one was treated with ustekinumab, and the second was without medical treatment). Importantly, at visit 6, anti-N was positive in 27 participants, of whom 21 were patients with IBD (10 were treated with anti-TNFα, and 11 were in the non-anti-TNFα group) and 6 were HCs. All infected subjects were asymptomatic and not aware of having been infected.

Interestingly, all 24 patients declining the third vaccine dose were not infected by SARS-CoV-2 (negative anti-N). Their serologic response at nine months after the first vaccine dose was significantly lower, as expected (GMC of 454 compared to 14559 after vaccination). Of these 24 subjects, 13 were HCs, and 11 were patients with IBD, four of whom were treated with anti-TNFα and seven of whom were not. Anti-S levels were significantly lower in the anti-TNFα group compared to both the non-anti-TNFα and HC groups (GMCs: 78 (CI:22–281), 657 (CI:336–1287), and 621 (CI:229–1683), respectively), similarly to the serology one month and six months after two vaccine doses [3,10].

### 3.3. Additional Predictors of Lower Vaccine Responses

Next, we evaluated different predictors of lower vaccine responses. Anti-N-positive and previously infected patients were excluded. In univariate analysis, we noticed that in addition to anti-TNFα treatment, older age, a longer interval between the second and third vaccine doses, and a longer interval between the third vaccine dose and visit 5 were also associated with lower serologic response at both visits 5 and 6 (Tables S1 and S2). At visit 5, only age, the interval between vaccination and visit 5, and anti-TNFα treatment remained significant in a multivariate linear regression model, while at visit 6, only anti-TNFα treatment remained significant (Table 2).

### 3.4. Anti-TNFα Drug Levels Do Not Affect Serologic Responses

One month after the third vaccine dose, we measured anti-TNFα drug levels and antibodies. Notably, measurement was performed at the time of serologic assessment rather than later. No correlation between anti-TNFα drug levels or antidrug antibodies and serologic response was observed (Spearman’s correlation). We further asked whether lower responses in patients treated with anti-TNFα were affected by the time interval between anti-TNFα drug administration and vaccination; however, timing of drug administration did not affect the serologic response. These results are consistent with the lack of correlation between drug levels after two vaccine doses and serologic response [10].

### 3.5. Vaccine Is Safe in Patients with IBD and Is Not Associated with Increased IBD Activity

One of the most concerning issues for both patients and physicians is the vaccine safety profile. We evaluated AEs using questionnaires and IBD activity using clinical and laboratory variables. A month after the third vaccine dose, no serious AEs were registered. AEs were more common after the third vaccine dose than after the second dose (81% vs. 76%). The most common AEs were local pain (81.5%), fatigue (44.4%), and headache (28.5%). AEs were comparable between the three study groups (Table S3).

Finally, IBD activity, as evaluated using HBI and SCCAI questionnaires, showed that 70% (88/125) of all patients with IBD were in clinical remission a month after the third vaccine dose, and 30% (37/125) had active disease (Figure S2, Table S4). We found that IBD activity was comparable in patients treated with anti-TNFα or not in the month following the third vaccine dose (*p* = 0.495 and *p* = 0.566 for HBI and SCCAI, respectively, using Pearson’s correlation). Neither CRP levels nor WBC counts increased following vaccination in either group. These results remained similar at visit 6 (Figure S2) and are consistent with reports regarding disease activity after the first two vaccine doses [10].

### 3.6. Lower Serologic Response Predicts Infection

The Omicron wave in Israel spanned from November 2021 to January 2022. Infection was recorded in a one- to four-month followup period after the third vaccine dose (after visit 6). Infection rates were proactively evaluated by reviewing subject records, specifically COVID-19 PCR tests and institutional COVID-19 antibody assays, and by calling subjects to identify home-evaluated infection. It is important to note that all infected participants had a mild COVID infection response that did not result hospitalization.

**Figure 3:**
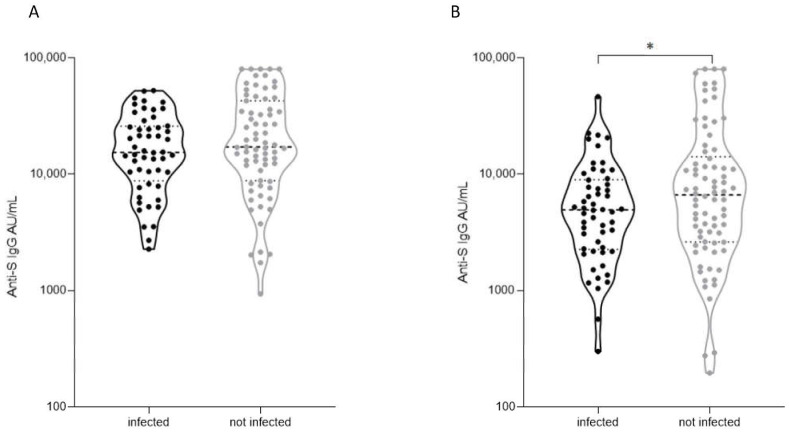
Association between serologic response and infection. (**A**) Serologic responses at visit 5 of subgroups among all participant stratified according to their infection status: infected (50, black) and not infected (64, grey). (**B**) Serologic responses at visit 6 of subgroups among all participant stratified according to their infection status: infected (54, black) and not infected (78, grey); black solid lines denote the median, and dashed lines denote IQR 25–75. Statistical analysis was carried out using unpaired Student’s *t*-test, *—*p* < 0.05. Anti-N-positive patients were excluded from this analysis, and only volunteers with three vaccine doses were included.

Out of 137 subjects who were vaccinated with three doses (and were anti-N-negative at both visits 5 and 6), 58 (42%) were infected during the followup period (see Figure 1), as documented by either antigen or PCR tests, and 79 were not. Importantly, anti-S levels at visit 6 of those who were infected during the followup period were significantly lower than those who were not infected (4770 (3709–6134) and 7483 (5649–9913), respectively; *p* = 0.0105, Figure 3). No such association was found at visit 5.

## 4. Discussion

Anti-TNFα is a mainstay therapy for patients with IBD. It is associated with higher rates of susceptibility to infection and with lower vaccination immune response [20,21,22,23]. During the COVID-19 pandemic era, patients with IBD were encouraged to vaccinate, despite their exclusion from phase 3 trials. In previous studies, we reported that while antibody responses to BNT162b2 vaccine developed in the anti-TNFα group, it was significantly lower compared to that in patients in the non-anti-TNFα and HC groups [3,10]. Furthermore, antibody response decays faster in this group of patients. Therefore, we proposed that a third vaccine dose might be crucial for patients with IBD treated with anti-TNFα [3,10,11,13,24]. In the current study, we investigated the serologic and clinical response to a third vaccine dose in patients with IBD either treated with anti-TNFα or not compared to HCs. As attenuated response to COVID-19 vaccines in patients treated with anti-TNFα was reported in other chronic inflammatory diseases [25,26], our results may have important clinical implications in these conditions as well.

We show that patients with IBD—regardless of treatment—and HCs became seropositive and had a robust increase in anti-S levels one month after the third BNT162b2 vaccine. Interestingly, patients with IBD treated with anti-TNFα had the steepest increase in anti-S levels after the third vaccine compared to the other groups.

Importantly, besides anti-TNFα treatment, older age and a longer interval between the second and third vaccine doses were independent predictors of lower serologic response. Older age is a risk factor for severe COVID-19 and poor vaccine effectiveness. These data further support the need for patients treated with anti-TNFα agents, specifically older ones, to receive an early booster vaccine and perhaps be relevant candidates for a fourth vaccine dose.

Next, we show that one month after the third vaccine dose, there are no documented serious AEs, similar to what we reported after the first two BNT162b2 doses [10]. AEs included local pain, headache, and fever, as described in previous studies [27,28]. The rate of AEs was higher one month after the third vaccine dose than one month after the second dose and comparable between all study groups (Table S3). In contrast, Dalin and colleagues [29] reported that after the third vaccine dose, there were fewer AEs compared to after the second vaccine dose. However, in both studies, the difference was not significant, supporting the overall safety of the third vaccine.

A major concern was the long-term safety and potential for immune-mediated inflammatory disease activity related to SARS-CoV-2 vaccines. Reassuringly, IBD activity remained stable one month after the third vaccine dose, regardless of patients’ medical treatment, as measured by clinical activity or inflammatory indices. Although approximately 30% of patients had active disease before the third vaccine dose, their disease status was not affected by vaccination. Therefore, a third vaccine dose can and should be recommended for patients with IBD, regardless of disease activity.

We recently reported that anti-TNFα treatment was unrelated to impaired responses to the first two vaccine doses [10]. Here, we also addressed vaccine timing relative to anti-TNFα drug administration and showed that there is no correlation between anti-S levels (after the third dose) and the interval between anti-TNFα administration.

Importantly, while seropositivity per se is considered crucial for protection from infection, here, we show that even in seropositive subjects, anti-S levels are important in determining infection susceptibility. Specifically, higher serologic response was associated with more protection from infection; in the 42.3% subjects who were infected despite receiving a third vaccine dose and being seropositive, the mounting serologic response was significantly lower than in subjects who were not infected (Figure 3). Previous studies also described breakthrough infections after the booster shot, despite a serologic response above the threshold, but they did not show an association between breakthrough infection and serologic response [30,31]. Moreover, in our cohort, we systematically monitored SARS-CoV-2 infections by consecutive testing for anti-N antibodies and can therefore inform on the protective effect of IgG anti-S antibodies induced exclusively by the third vaccine dose without the contribution of natural infection (no hybrid immunity). An Israeli study investigated the characteristics of patients who were fully vaccinated and had a significant vaccine breakthrough that led to hospitalization. They found an association between comorbidities and severity of disease, without an association with serologic response [32]. 

This is a prospective study assessing the dynamics of serologic responses to three vaccine doses in patients with IBD stratified by therapy compared to non-IBD controls. Patient persistence is a strong advantage of this study, as the majority of patients recruited before the first vaccine dose remained for followup for three vaccine doses. Importantly, in addition to following 51 patients with IBD treated with anti-TNFα, we also followed 74 patients with IBD untreated with anti-TNFα, whether treated with other IBD therapies or untreated. Thus, we were able to report the responses to three vaccine doses in these subgroups. In addition to detecting disease activity using clinical scores and inflammatory indices, we were able to provide prospective, reassuring data regarding the lack of serious AEs and IBD exacerbation (Figure S2). Additionally, the lack of correlation between the timing of anti-TNFα administration and drug levels encourages clinicians to follow the original treatment plan.

We acknowledge several limitations, mainly related to the baseline cohort presented in our previous report [10]. These include small numbers of patients treated with steroids and immunomodulators, a limitation relevant to most other reports in IBD focusing mainly on anti-TNFα therapies. Another potential limitation is that the number of patients with CD is higher than that of UC Additional limitations include differences in gender ratios in the IBD and HC groups at baseline. Finally, a single commercial kit was used to measure anti-S (Abbott SARS-CoV-2 IgG II kit), while several others are available. However, even if there are minor differences in the measurements between the available kits, the clear significant findings probably would not have been affected.

## 5. Conclusions

This prospective study demonstrates that a third dose of the BNT162b2 vaccine is efficacious and safe in patients with IBD. However, patients treated with anti-TNFα had a significantly lower serological response compared with patients not treated with anti-TNFα. The lack of correlation between anti-TNF-α drug levels and immune response suggests that there is no need to alter the timing of vaccination compared to anti-TNF-α drug administration. 

The large increase in anti-S values between the second and third doses of vaccine suggests that the third dose is important in patients receiving anti-TNFα therapy. A higher serological response predicts better protection against COVID-19. Furthermore, the significant reduction in anti-S levels in response to anti-TNFα treatment may indicate the benefit of a fourth dose of vaccine for IBD patients.

## Figures and Tables

**Table 1:** Baseline demographic characteristics of participants.

	Visit 5				Visit 6			
Characteristic	Anti-TNFα *n* = 53	Non-Anti-TNFα *n* = 72	HC *n* = 73	*p* Value	Anti-TNFα *n* = 46	Non-Anti-TNFα *n* = 66	HC *n* = 76	*p* Value
Mean age, years (SD)	39.8 (14.7)	38.6 (14.9)	39.4 (12.5)	0.89	38.4 (14.0)	37.1 (13.1)	38.2 (11.1)	0.838
Female, *n* (%)	20 (37.7)	31 (43.1)	51 (69.9)	<0.001	13 (28.3)	31 (47.0)	54 (71.1)	<0.001
Origin, *n* (%)								
Ashkenazi	28 (52.8)	36 (50.0)	40 (54.8)	0.845	22 (47.0)	29 (43.9)	37 (48.7)	0.842
Non-Ashkenazi	25 (47.2)	38 (50.0)	33 (45.2)		24 (52.2)	37 (56.1)	39 (51.3)	
Mean BMI, kg/m^2^ (SD)	25.4 (4.4)	23.8 (5.3)	25.4 (5.2)	0.112	25.4 (4.1)	23.8 (5.3)	25.6 (5.2)	0.086
Smoking status, *n* (%)								
Present	5 (9.6)	8 (11.1)	5 (6.8)	0.408	3 (6.5)	7 (10.6)	6 (7.9)	0.373
Past	2 (3.8)	3 (4.2)	0 (0)		1 (2.2)	3 (4.5)	0 (0)	
No	46 (86.8)	61 (84.7)	68 (93.2)		42 (91.3)	56 (84.8)	70 (92.1)	
Comorbidities ^a^, *n* (%)	5 (9.4)	5 (6.9)	4 (5.5)		8 (17.4)	8 (12.1)	8 (10.5)	
IBD phenotype, *n* (%)								
CD	43 (81.1)	40 (55.6)	-----	0.004	38 (82.6)	40 (60.6)	-----	0.013
UC	7 (13.2)	26 (36.1)	-----	0.004	5 (10.9)	22 (33.3)	-----	0.007
IPAA	2 (3.8)	4 (5.6)	-----		2 (4.3)	3 (4.5)	-----	
IBD-U	1 (1.9)	2 (2.8)	-----		1 (2.2)	1 (1.5)	-----	
Disease activity ^b^, *n* (%)								
Remission	34 (65.4)	54 (75.0)	-----	0.316	34 (75.6)	47 (72.3)	-----	0.827
Active	18 (34.6)	18 (25.0)	-----		11 (24.4)	18 (27.7)	-----	
Current medication, *n* (%)								
IFX	29 (54.7)	-----	-----		28 (60.9)	-----	-----	
ADA	22 (41.5)	-----	-----		17 (37.0)	-----	-----	
Cetrolizumab-Cimzia	2 (3.8)	-----			1 (2.2)	-----		
Vedolizumab	-----	18 (25.0)	-----		------	15 (22.7)	-----	
Ustekinumab	-----	10 (13.9)	-----		------	8 (12.1)	-----	
5-ASA	5 (9.8)	19 (26.4)	-----		3 (6.5)	17 (25.8)	-----	
Steroids	2 (3.8)	3 (4.2)	-----		------	2 (3.0)	-----	
Immunomodulators ^c^	8 (15.1)	1 (1.4)	-----		11 (23.9)	3 (4.5)	-----	
JAK inhibitor	-----	4 (5.6)	-----		-----	4 (6.1)	-----	
No medical treatment	-----	20 (27.8)	-----		-----	23 (34.8)	-----	

^a^ The main comorbidities were asthma, diabetes mellitus, and high blood pressure, in addition to fatty liver disease, celiac, hypothyroidism, ankylosing spondylitis, and prostate cancer. ^b^ Disease activity was quantified clinically by validated questionnaires. ^c^ Including 6-mercatopurine, azathioprine, and methotrexate. Abbreviations: HC = healthy control, BMI = body mass index, CD = Crohn’s disease, UC = ulcerative colitis, IBD-U = IBD—unclassified, IPAA = ileal pouch–anal anastomosis, IFX = infliximab, ADA = adalimumab, 5-ASA = 5-aminosalicylic acid.

**Table 2:** Multivariate analysis at visit 5 (**A**) and visit 6 (**B**).

**A**			
**Variable**		**B (95% CI)**	***p* value**
Treatment	Anti-TNFα	−0.482 (−0.785 to −0.180)	0.002
	Non-anti-TNFα	0.007	0.928
	HC	reference	
Age (years)		−0.013 (−0.023 to −0.003)	0.008
Δ dose 3 and visit 5 (days)		−0.021 (−0.03 to −0.011)	<0.001
Δ dose 2 and dose 3 (days)		−0.010	0.886
**B**			
**Variable**		**B (95% CI)**	***p* value**
Treatment	Anti-TNFα	−0.789 (−1.255 to −0.322)	0.001
	Non-anti-TNFα	0.092	0.288
	HC	Reference	

Δ means the time from the third dose to visit 5, and the time from the second to the third dose.

## Data Availability

The data underlying this article are available in the article and in its online Supplementary Materials.

## Supplementary Materials

The following supporting information can be downloaded at: https://www.mdpi.com/article/10.3390/vaccines11071263/s1, Table S1: Univariate analysis at visit 5. Table S2: Univariate analysis at visit 6. Table S3: Specific adverse events one month after third vaccine dose. Table S4: Change in disease activity measured by the difference in questionnaire scores (HBI, SCCAI for patients with CD and UC, respectively). Figure S1: (A) Serologic responses in visits 5 to subgroups within non-anti-TNFα group stratified according to their medical treatment: no medical treatment (in black), 5-ASA (in dark grey), Vedolizumab (in light grey), other medical treatment which relatively small subgroups (Steroids, immunomodulators, ustekinumab and tofacitinib; in brown). Black solid lines denote median, dashed lines denote IQR 25-75. (B) Serologic responses in visits 6 to subgroups within non-anti-TNFα group stratified according to their medical treatment: no medical treatment (in black), 5-ASA (in dark grey), Vedolizumab (in light grey), other medical treatment which relatively small subgroups (immunomodulators, ustekinumab, tofacitinib and clinical study drug; in brown). Black solid lines denote median, dashed lines denote IQR 25-75. Figure S2: Disease activity during follow up. Activity was measured by validated questionnaires. Bars represent the average score of either HBI for CD or SCCAI for UC, stratified according to treatment (with and without anti-TNFα), after 1- and 3- months three vaccine doses (visit 5, black; visit 6, grey, respectively). Error bars denote SD. The difference between the groups was not significant using Independent-Samples Kruskal–Wallis Test.
