# Supplementary material for: Serologic Response and Safety after a Third Dose of the COVID-19 BNT162b2 Vaccine in Patients with Inflammatory Bowel Diseases
Source: Vaccines (Basel). 2023 Jul 20;11(7):1263. doi: 10.3390/vaccines11071263 (PMC10386081; doi:10.3390/vaccines11071263)
Supplement: Supplementary file 1 [file vaccines-11-01263-s001.zip › vaccines-2432804-supplementary.pdf]

Supplemental data:

Table S1: Univariate analysis at visit 5

Variable		n/N	GMC (95%CI)/Spearman's rho	P Value
Treatment	HC	73/197	17069 (14085-20684)	**0.020**
	Non-anti-TNFα	71/197	16594 (12977-21220)	
	Anti-TNFα	53/197	9709 (6985-13495)	
Non-anti-TNFα subgroups	Vedolizumab	17/124	24448 (15834-37748)	**0.037**
	5-ASA	18/124	10010 (6280-15953)	
	Other	69/124	11316 (8320-15391)	
	No treatment	20/124	17032 (12151-23872)	
Gender	Male	96/197	135353 (12305-19155)	0.461
	Female	101/197	13765 (11339-16709)	
Age (years)		194/197	Rho=-0.186	**0.009**
BMI (kg/m^2)		196/197	Rho=0.063	0.381
Diagnosis	CD	82/124	12924 (9825-16999)	0.976
	UC	33/124	13599 (9889-18700)	
	IBD-U	3/124	14037 (1115-176662)	
	IPAA	6/124	14439 (5412-38522)	
Hemoglobin value (g/dL)		179/197	Rho=0.025	0.743
WBC value (K/micl)		195/197	Rho=0.037	0.608
CRP value (mg/dL)		192/197	Rho=0.064	0.379
Δ dose 2 and dose 3 (days)		197/197	Rho=0.149	**0.037**
Δ dose 3 and visit 5 (days)		197/197	Rho=-0.260	**<0.001**

Table S2: Univariate analysis at visit 6

Variable		n/N	GMC (95%CI)/Spearman's rho	P Value
Treatment	HC	66/149	6509 (5086-8330)	**0.003**
	Non-anti-TNFα	52/149	8209 (5863-11493)	
	Anti-TNFα	31/149	3283 (1989-5418)	
Non-anti-TNFα subgroups	Vedolizumab	13/82	12687 (6816-23614)	**0.031**
	5-ASA	9/82	9703 (4456-21126)	
	Other	38/82	4001 (2464-6495)	
	No treatment	22/82	5585 (3299-9454)	
Gender	Male	69/149	6464 (4775-8751)	0.608
	Female	80/149	5840 (4522-7540)	
Age (years)		147/149	Rho=-0.137	0.099
BMI (kg/m^2)		147/149	Rho=0.134	0.106
Diagnosis	CD	59/83	5304 (3638-7732)	0.787
	UC	19/83	7593 (4556-12656)	
Hemoglobin value (g/dL)		146/149	Rho=0.128	0.124
WBC value (K/micl)		146/149	Rho=-0.078	0.349
CRP value (mg/dL)		143/149	Rho=0.128	0.127

Table S3: Specific adverse events one month after third vaccine dose

**Adverse events, n (%)**	**Anti-TNFα**	**Non-anti-TNFα**	**HC**
Pain at injection site	30 (78.9)	47 (81.0)	46 (83.6)
Swelling at injection site	8 (21.1)	8 (13.8)	6 (10.9)
Fever (>38°c)	4 (10.5)	9 (15.5)	1 (1.8)
Headache	14 (37.8)	17 (28.8)	12 (21.8)
Shivering	4 (10.8)	10 (16.9)	7 (12.7)
Nausea	2 (5.4)	3 (5.1)	4 (7.3)
Dizziness	3 (8.1)	8 (13.6)	4 (7.3)
Fatigue	21 (56.8)	28 (47.5)	18 (32.7)
Muscle soreness	4 (10.8)	12 (20.3)	6 (10.9)
Joints pain	3 (8.1)	5 (8.5)	1 (1.8)
Allergic reaction	-----	-----	1 (1.8)
Others[a]	3 (8.1)	9 (15.3)	10 (18.2)

[a]Others include = general weakness, flu-like symptoms, vaginal bleeding, lymph node swelling, cough, throat and ear pain, aphthous stomatitis.

Table S4: Change in disease activity measured by the difference in questionnaire scores (HBI, SCCAI for patients with CD and UC, respectively)

	**CD**	**UC**
Clinical improvement	32	11
Clinical worsening	6	1
No change	44	21

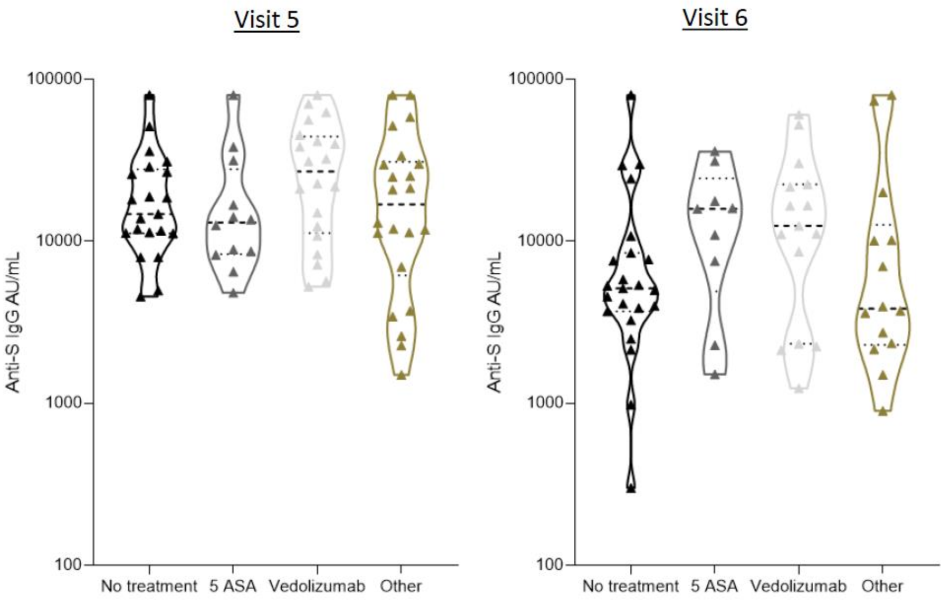

Figure S1: (A) Serologic responses in visits 5 to subgroups within non-anti-TNFα group stratified according to their medical treatment: no medical treatment (in black), 5-ASA (in dark grey), Vedolizumab (in light grey), other medical treatment which relatively small subgroups (Steroids, immunomodulators, ustekinumab and tofacitinib; in brown). Black solid lines denote median, dashed lines denote IQR 25-75. (B) Serologic responses in visits 6 to subgroups within non-anti-TNFα group stratified according to their medical treatment: no medical treatment (in black), 5-ASA (in dark grey), Vedolizumab (in light grey), other medical treatment which relatively small subgroups (immunomodulators, ustekinumab, tofacitinib and clinical study drug; in brown). Black solid lines denote median, dashed lines denote IQR 25-75.

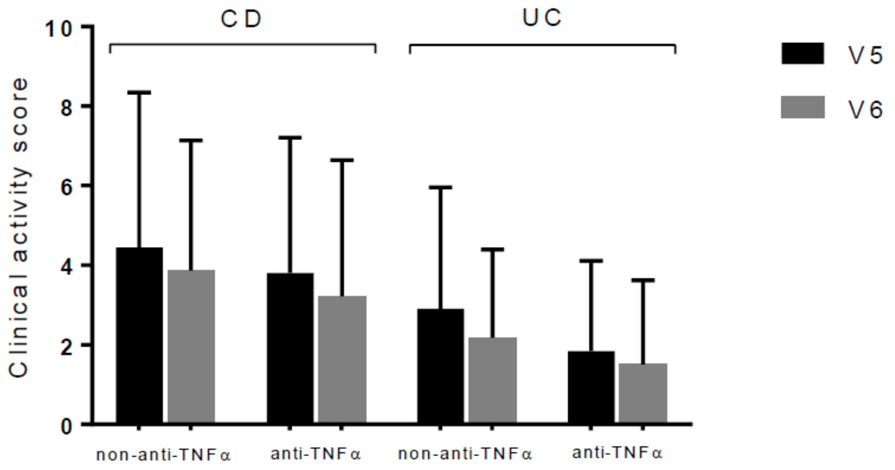

Figure S2: Disease activity during follow up. Activity was measured by validated questionnaires. Bars represent the average score of either HBI for CD or SCCAI for UC, stratified according to treatment (with and without anti-TNFα), after 1- and 3- months three vaccine doses (visit 5, black; visit 6, grey, respectively). Error bars denote SD. The difference between the groups was not significant using Independent-Samples Kruskal-Wallis Test.

Abbreviations: HBI=Harvey-Bradshaw Index; SCCAI=Simple Clinical Colitis Activity Index, UC=Ulcerative colitis, CD=Crohn's disease
